# Pulmonary embolism with thrombus in transit across a patent foramen ovale

**DOI:** 10.1093/omcr/omae091

**Published:** 2024-08-23

**Authors:** Sumedh Iyengar, Anton Stolear, Maxim Dulgher, Ashraf Ahmed, Evgeny Shkolnik, Stuart Zarich

**Affiliations:** Yale New Haven Health - Bridgeport Hospital Department of Internal Medicine, Bridgeport, CT 06610, United States; Yale New Haven Health - Bridgeport Hospital Department of Cardiology, Bridgeport, CT 06610, United States; Norwalk Hospital Department of Internal Medicine, Norwalk, CT 06856, United States; Yale New Haven Health - Bridgeport Hospital Department of Internal Medicine, Bridgeport, CT 06610, United States; Yale University School of Medicine Department of Cardiology, New Haven, CT 06510, United States; Yale University School of Medicine Department of Cardiology, New Haven, CT 06510, United States

**Keywords:** thromboembolism-in-transit, impending paradoxical embolus, surgical thrombectomy, clot-in-transit, patent foramen ovale, pulmonary embolism

## Abstract

Thromboembolism-in-transit, specifically impending paradoxical embolism (IPDE), is a rare and life-threatening condition with limited reported cases. We present a case of a 51-year-old male with obstructive sleep apnea, initially diagnosed with deep vein thrombosis and pulmonary embolism. Further evaluation revealed a saddle pulmonary embolus extending into the right atrium, straddling a patent foramen ovale (PFO), confirmed by transesophageal echocardiogram. Despite a critical left anterior descending coronary artery stenosis, surgical thrombectomy, PFO closure, and coronary artery bypass grafting were successfully performed. Thromboembolism-in-transit poses diagnostic challenges, and there is a lack of consensus on the optimal treatment strategy. Surgical interventions, including embolectomy and PFO closure, have shown promise, while thrombolytic therapy remains controversial. This case underscores the importance of tailored management in the absence of standardized guidelines, emphasizing the need for further research to establish evidence-based protocols for this uncommon but potentially fatal condition.

## Introduction

Paradoxical embolism is a rare phenomenon, accounting for less than 2% of all arterial emboli. Impending paradoxical embolism (IPDE) or thromboembolism-in-transit, in which a thrombus straddles an interatrial defect, is an even rarer diagnosis that is usually made incidentally [1]. It is associated with high mortality (30-day mortality of 18.4%, with 62.5% of deaths during the first 24 h of presentation) [2]. While the severity and complications of thromboembolism-in-transit are recognized, the disease entity is still rarely considered and remains under-reported [3]. The true prevalence of thromboembolism-in-transit is unknown as its proven clinical diagnosis remains challenging, making it a presumptive diagnosis in most cases. We discuss a case of deep vein thrombus with pulmonary embolism (PE) -in-transit.

## Case presentation

**Figure 1:**
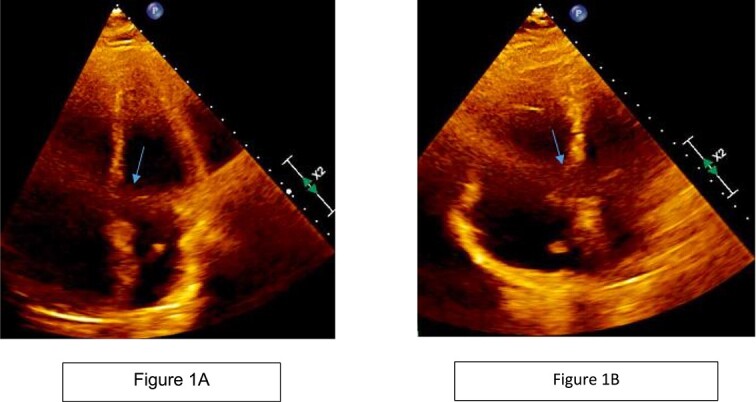
Transthoracic echocardiogram raising suspicion for a mobile mass in each atrium.

**Figure 2:**
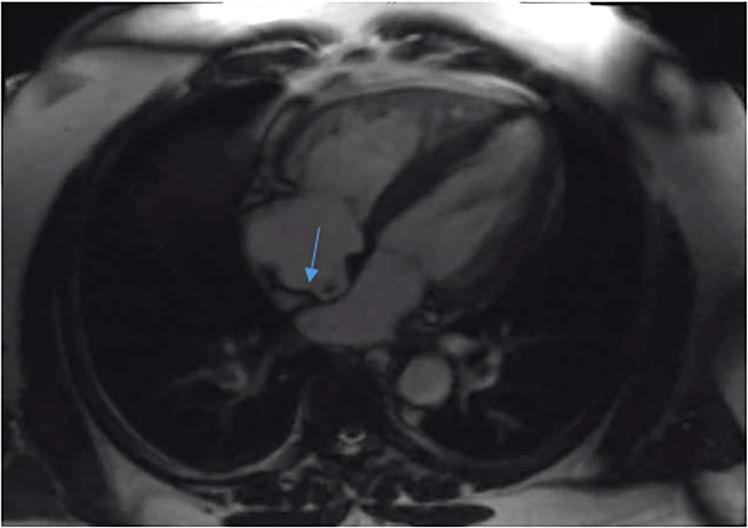
Cardiac magnetic resonance imaging showing a filling defect in the right atrium close to the interatrial septum.

**Figure 3:**
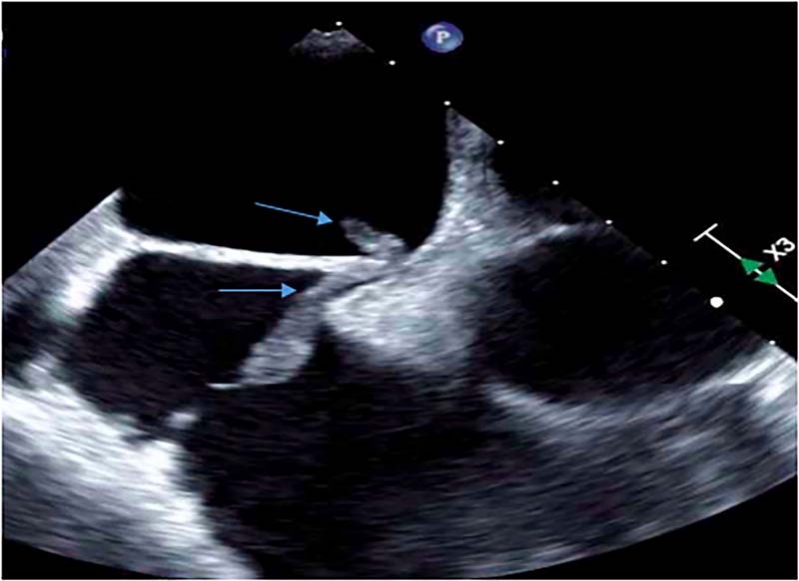
Transesophageal echocardiogram confirmed a PFO with a large thrombus seen in transit from the right atrium to left atrium.

A 51-year-old African American male with obstructive sleep apnea presented with progressive dyspnea and palpitations for 1 day. He was hemodynamically stable with oxygen saturation 95% on room air. Physical exam was notable for moderate respiratory distress and mild right lower extremity edema. EKG showed sinus tachycardia without signs of ischemia. Troponin T and D-dimer were elevated at 0.05 ng/ml (reference range: 0–0.01 ng/ml) and 10.13 mg/l (reference range < 0.50 mg/l) respectively. Computed Tomography Angiography (CTA) chest was done, which showed a saddle pulmonary embolus, dilated main pulmonary artery, right ventricular enlargement, and signs of right ventricular strain. It was graded as a sub-massive pulmonary embolism. Bedside transthoracic echocardiography showed an ejection fraction of 55%–60% with right ventricular enlargement and strain with a positive McConell sign. A heparin drip was started and the patient was taken for pulmonary artery catheter directed thrombolysis (Ekosonic Endovascular system directed thrombolysis). A comprehensive transthoracic echocardiogram (TTE) the following day raised suspicion for a mobile thrombus in each atrium (Fig. 1). A lower extremity ultrasound further revealed a right popliteal DVT as the likely source of the emboli. CTA chest did not reveal thrombi in the atria. Cardiac magnetic resonance imaging showed a filling defect in the right atrium close to the interatrial septum (Fig. 2). Transesophageal echocardiogram (TEE) was done for better visualization of the atria, and showed the presence of patent foramen ovale (PFO) with large thrombus seen in transit from right atrium to left atrium (Fig. 3). There was no clot seen in the left atrial appendage. The bubble study was not performed. Cardiothoracic surgery was consulted, who recommended performing a surgical thrombectomy after placement of an inferior vena cava filter and pre operative coronary angiogram. Left heart catheterization revealed critical stenosis of ostial left anterior descending (LAD) coronary artery. Cardiac surgery was performed. A large clot was seen straddling the PFO with some residual clot in the inter-atrial septum that could not be removed. Thus, the inter-atrial septum was excised and repaired in its entirety with a bovine pericardial patch. The left atrial clot and the clot that was residing in the PFO was removed and sent for histopathalogical examination. Concurrent left internal mammary artery to LAD artery grafting was also performed. The patient had an uneventful post-operative course and recovered without complications.

## Discussion

Thromboembolism-in-transit across a patent foramen ovale, also referred to as impending paradoxical embolism (IPDE), is an infrequent occurence despite the high prevalence of PFO at 26% [2]. The abrupt rise in pulmonary arterial pressure may contribute to the migration of the thrombus across the atrial septum to the systemic circulation. Thromboembolism-in-transit is associated with high mortality (30-day mortality of 18.4%, with 62.5% of deaths during the first 24 h of presentation), and morbidity [4]. Furthermore, the presence of obstructive sleep apnea in this patient, which has been shown to be an independent risk factor for venous thromboembolism, likely puts him at a greater risk of developing pulmonary embolism [5].

Currently, there are no definitive guidelines for managing thromboembolism-in-transit. No controlled clinical trials have been conducted to establish treatment protocols for impending paradoxical embolism (IPDE) [6]. Potential therapeutic approaches include surgical extraction, systemic anticoagulation, and thrombolysis, but consensus on the optimal treatment strategy, especially for incidentally discovered cases, is lacking [2]. Thrombolysis carries a high risk of recurrent systemic embolization. A study by Myers et al comparing thrombolysis and thromboembolectomy for IPDE patients showed a nonsignificant trend towards improved survival and reduced systemic embolization and mortality in those who underwent thromboembolectomy [3]. Farfel et al, in a study involving 49 patients with right atrial thromboemboli, found a 15% mortality rate in those treated with surgical embolectomy and a 50% mortality rate in those treated medically with thrombolytic agents, anticoagulants, or supportive therapy. Of these, 3 were treated surgically and survived and 1 was treated unsuccessfully with streptokinase. The authors suggested surgical management as the preferred approach for IPDE [4], a view supported by Armstrong et al [7]. Loscalzo et al recommend intracardiac embolectomy and inferior vena cava interruption [8].

Meacham et al, conducted a literature review of 30 cases of IPDE and concluded that the treatment of IPDE should consist of initial systemic heparinization to reduce the immediate threat of further embolic phenomenon while awaiting emergent intracardiac surgical embolectomy with PFO closure [9]. Surgical embolectomy is an effective treatment for removing the heart thrombus straddling the interatrial septum and it makes it possible to directly close the PFO. Thrombolytic therapy can be used in patients with a high risk related to surgery. There have also been newer case reports exploring the use of Flowtriever retrieval/aspiration system as a method of percutaneous catheter directed mechanical thrombectomy which has shown significant improvement of left ventricular/right ventricular ratio with a good safety profile [10]. Regarding primary prevention, percutaneous or surgical defect closure of PFO has not been considered previously for primary prevention of IPDE. However, PFO closure should be done in patients with IPDE or recurrent paradoxical embolus [4].

The current variety of treatment options of IPDE shows the need for robust evidence to provide patients with optimal care. Although it has been proposed previously, evaluating the requirement for a multicenter randomized trial is essential.
